# Lymphatic Endothelial Cell in Endemic Bancroftian Filariasis: A Focus on the Lymphatics of the Tunica Vaginalis Testis

**DOI:** 10.1155/2018/5134670

**Published:** 2018-05-16

**Authors:** Jose Figueredo-Silva, Joaquim Norões, Gerusa Dreyer

**Affiliations:** ^1^Núcleo de Ensino e Pesquisa em Patologia, Faculdade de Ciências Médicas (FACIME), Universidade Estadual do Piauí, Teresina, PI, Brazil; ^2^Departamento de Cirurgia, Centro de Ciências da Saúde, Universidade Federal de Pernambuco, Recife, PE, Brazil; ^3^Núcleo de Ensino, Pesquisa e Assistência em Filariose (NEPAF), Hospital das Clínicas, Universidade Federal de Pernambuco, Recife, PE, Brazil; ^4^Organização Não Governamental Amaury Coutinho para Doenças Endêmicas e Tropicais, Recife, PE, Brazil; ^5^Centro de Pesquisas Aggeu Magalhães, Fundação Oswaldo Cruz (FIOCRUZ), Recife, PE, Brazil

## Abstract

**Background:**

In endemic areas, lymphangiectasia is the fundamental alteration to live* Wuchereria bancrofti* adult worms which, in adult males, are usually found in the lymphatic vessels of the spermatic cord; accordingly, hydrocele/filaricele is the most common clinical manifestation of bancroftian filariasis. The pathogenic role of the lymphatic endothelial cells (LECs) and the status of mesothelial cells (MCs) samples of the parietal layer (PL) of the tunica vaginalis testis were examined.

**Methods:**

The PL of thirty-two patients, excised for different reasons, was examined by histology and immunohistochemistry using the D2-40 monoclonal antibody for identification of LECs and CK-7 antibody for recognition of mesothelial cells (MCs).

**Results:**

The most important findings were (a) marked lymphangiectasia, especially in hydroceles with minor evolution time; (b) the first report of lymphatic stomata and submesothelial lacunae in filarial acute hydrocele; (c) the likely participation of LECs in filarial granuloma; (d) the potential phenotypic transition of LECs into myofibroblasts in severe chylocele; and (e) mesothelial reactive hyperplasia, a hallmark of filaricele, varying in intensity from mild to severe, sometimes mimicking a mesothelial neoplasia.

**Conclusion:**

The data suggest that LECs have an active role in the pathogenesis of bancroftian hydrocele and, possibly, in other clinical forms of lymphatic filariasis.

## 1. Introduction

Lymphatic vessels (LVs) have historically been viewed as inert drainage system for fluid and immune cells, but this merely passive concept is increasingly being revised as new functions of lymphatic endothelial cells (LECs) are identified. They have been shown to play active roles in controlling their transport functions and in directly communicating with immune cells to modulate their immediate and downstream function. A growing body of evidence is demonstrating how LECs help shape both innate and adaptive immune responses through (a) expression of multiple cytokines, adhesion molecules, and inhibitory receptors; (b) scavenging and processing antigens for direct presentation to T cells or modulating the activity of professional antigen-presenting cells (APCs); and (c) actively regulating fluid and solute transport functions in response to inflammatory signals [1]. In addition, LVs within different organs and in different physiological and pathological processes show a remarkable plasticity and heterogeneity, reflecting their functional specialization. LECs of diverse organs were recently shown to have alternative developmental origins, which may contribute to the development of the diverse lymphatic vessel and endothelial functions seen in the adult individuals. Inflammation and fibrosis by malfunction of LVs are associated with the pathogenesis of lymphedema [2]. Lymphangiogenesis is also linked to many inflammatory diseases and tumor-related lymphangiogenesis is an important mechanism by which metastatic cells disseminate to lymph nodes and distant organs [3].

Given their role in health and disease, it is no surprise that they have a relevant role in the pathogenesis of filariasis caused by the lymphatic-dwelling nematodes* Wuchereria bancrofti*,* Brugia malayi,* and* B. timori*. Integrating clinical, parasitological, surgical, therapeutic, ultrasonographic, and histopathological data, Dreyer et al. [4] maintain that in long-term residents in endemic areas the pivotal event in bancroftian filariasis (BF) is lymphangiectasia caused by live adult worms (AWs) with no apparent inflammation in the vessel wall. Lymphatic dilation develops in the vicinity of AWs nests but is not restricted to the exact segment of lymphatics where the AWs inhabit [5] and tends to progress in the presence of living AWs [6]. These data suggest that lymphangiectasia will progress and would be mediated by soluble products excreted or secreted by the parasite acting on the LECs. The death of the AWs, whether spontaneous or as a result of treatment with diethylcarbamazine citrate (DEC), interrupts the noninflammatory phase of lymphatic dilatation [4]. In its very early phase, the inflammatory process is nonspecific, but soon granulomas are formed around lethally injured AWs. The cellular composition and granuloma kinetics are described in detail elsewhere [7].

In males living in endemic areas,* W. bancrofti* AWs are typically found in the LVs of the spermatic cord and juxta testicular regions [6, 8, 9]. This may explain why hydrocele remains the most common clinical manifestation of BF even after the Global Programme to Eliminate Lymphatic Filariasis has completed 13 years of operation through 2012 with mass drug administration as the core strategy in 55 of 73 endemic countries [10]. The pathogenesis of acute and chronic filarial hydrocele is discussed in detail elsewhere [11, 12]. In short, acute filarial hydrocele is a consequence of acute interruption of lymph flow from the tunica vaginalis testis (TVT), caused by filarial granulomas (corresponding to formation of palpable nodules detected by physical examination of intrascrotal contents). The major mechanism of chronic filarial hydrocele, the so-called* filaricele*, is the presence of intrascrotal lymphatic fistulae which results in the gradual accumulation of a straw colored “filarial hydrocele fluid,” a combination of lymph fluid and transudate in different proportions [12].

In this investigation, immunohistochemistry using the D2-40 monoclonal IgG mouse antibody, which recognizes podoplanin, a 38-kDa transmembrane glycoprotein expressed on LECs, was used to better characterize the lymphatic network of the parietal layer (PL) of the TVT from patients born and living in a BF endemic area. The antibody has been reported to be the most specific and the most sensitive marker for detecting LVs and has been widely used in paraffin-embedded material [13, 14]. As podoplanin can also be expressed in normal and neoplastic mesothelial cells (MCs) [15], immunohistochemistry for cytokeratin 7 (CK7), which marks MCs, but not the lymphatic endothelium [16], was also used. To our knowledge, there are no published studies of the PL lymphatic network using this approach.

## 2. Materials and Methods

This retrospective study was conducted at the Núcleo de Ensino, Pesquisa e Assistência em Filariose (NEPAF), Hospital das Clínicas, Universidade Federal de Pernambuco, Recife, Pernambuco, and at the Núcleo de Ensino e Pesquisa em Patologia, the Universidade Estadual do Piauí, Teresina, Piauí, Brazil. The surgeries occurred between March 1994 and March 2006 as part of a larger comprehensive study on different aspects of urological manifestation of the BF and on the AWs themselves. Several publications resulted from the study, some of which are quoted here [4, 6, 9, 11, 17–20]. The study was approved by the Ethics Committee of Hospital das Clinicas at Federal University, Pernambuco, Brazil. Before surgery, all patients signed an informed consent form and the study was conducted in accordance with the principles of the Declaration of Helsinki and the guidelines on Good Clinical Practice.

### 2.1. Tissue Specimen Selection

Hematoxylin and eosin-stained slides of the PL removed for different reasons archived in NEPAF were reviewed and selected initially according to the following general inclusion criteria obtained from patients' medical records: (a) to have microfilaria (Mf) investigation in blood (Mf/Ml) collected before surgery and in fluid from vaginal testicular cavity collected per surgery; (b) to have the total fluid volume measurement collected during surgery from vaginal testicular cavity; (c) to have physical examination, including scrotal contents before surgery, and before and after antifilarial treatment, if this was the case; (d) to have intrascrotal ultrasound assessment with 7.5 MHz probe before surgery with the information about presence/absence: of lymphangiectasia; living adult worms and abnormal/normal presence of fluid in tunica vaginal testicular cavity; (e) to have received or not antifilarial treatment before surgery; (f) to not have ipsilateral inguinal hernia, history of past ipsilateral inguinal hernia surgical repair, intrascrotal surgery (including hydrocele repair, vasectomy, and varicocelectomy), or past history of ipsilateral bacterial infection; (g) presence/absence of lymphatic fistula detected per surgically generating a straw colored fluid or milk fluid; (h) to be born and have lived the entire life in endemic area; and (i) to have paraffin blocks with enough material for additional slides. Among a total of three hundred and forty-two surgical specimens, thirty-two most representative cases were selected. The paraffin blocks were retrieved and additional slides were obtained for conventional histology and immunohistochemistry.

### 2.2. Histology and Immunohistochemistry

Formalin-fixed, paraffin-embedded archival tissue blocks of specimens of the PL of the tunica vaginalis testis were used. There were 3–5 fragments per block. Four 5 *μ*m adjacent sections were obtained: two were stained with hematoxylin-eosin (HE) and the two remaining were used for CK-7 and D2-40 for immunohistochemistry. After deparaffinization and rehydration in graded alcohol series, the monoclonal antibodies D2-40 (Dako Corporation, Carpinteria, CA, USA; dilution 1 : 200) and anti-CK 7 (Dako, dilution 1 : 1000) were used, respectively. Antigen retrieval was obtained for the D2-40 test by boiling at 95–100 C in moist heat steamer for 60 minutes, and for CK-7 by conventional microwave oven for 15 minutes. Endogenous peroxidase was blocked by treatment with 3% hydrogen peroxide (H2O2) for 5 min. Immunohistochemistry for the D2-40 was performed using the EnVision System (Dako) in which the anti-mouse secondary antibody and detection system were incubated in a single step reaction for 45 minutes at room temperature. Sections for CK-7 were incubated with antimouse immunoglobulin biotinylated secondary antibody for 16 min at 37°C, followed by treatment with the avidin-biotin-horseradish peroxidase complex. The final revelation of the antibodies was performed by the peroxidase reaction visualized by staining with DAB [(3-3)-tetrahydrochloride diaminobenzidine)], counterstained with Mayer hematoxylin. Sections of a thyroid lymphangioma, processed similarly to samples of the PL, were used as positive control. All slides were examined by using an Olympus (CX31, YS100 model) microscope equipped with an Olympus SC20 digital camera. The diameters of the D2-40 stained lymphatic vessels were assessed by using the Image-J®, a public domain, Java-based image processing program developed at the National Institutes of Health (Bethesda, MD, USA). In order to evaluate, as accurately as possible, the variation in lymphatic diameter, only traverse or longitudinal segments of the vessels were surveyed.

## 3. Results

### 3.1. Groups

The following groups were characterized: (1)* control *(4 cases): symptomless individuals from which living AWs nonsensitive to antifilarial treatment were removed [17–20]; (2)* patients with dead AWs in the PL* (1 case); (3)* acute filarial hydrocele* (3 cases): patients with fluid accumulation in the tunica vaginalis cavity which appears within a few days after a nodule formation and resolves spontaneously up to 18 months [11]; (4)* filaricele* (20 cases): patients with chronic accumulation in the tunica vaginalis cavity of fluid composed of a combination, in different proportion, of transudate and nonmilky lymph from ruptured dilated lymphatic vessels [12]. The cases of this group were distributed in grades according to the intensity of the MCs hyperplasia: grade 0: MCs confined to the free surface; grade 1: MCs present in the submesothelial and not surpassing a third of the wall thickness; grade 2: MCs reach two-thirds of the wall; and grade 3: MCs are found in whole thickness of the wall; and (5)* chylocele *(4 cases): patients with chronic accumulation in the tunica vaginalis cavity of milky fluid as a result of lymph rich in chylomicrons from ruptured dilated lymphatic vessels [12]. The overall information of the cases is shown in Table 1. The Mf search in the fluid was negative irrespective of active infection status in all patients in the present study. All lymphangiectasia identified by ultrasound was confirmed per surgery.

### 3.2. Histopathology and Immunohistochemistry

**Figure 1:**
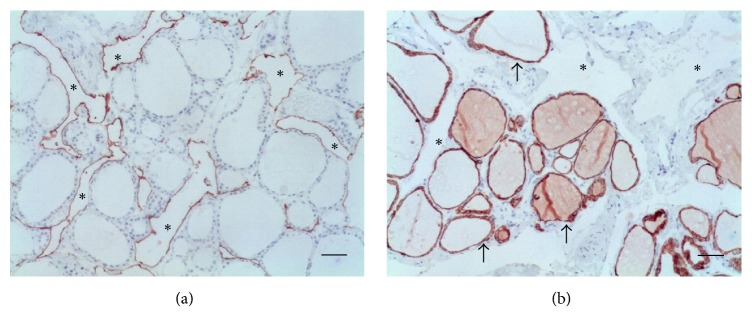
Thyroid lymphangioma. (a) Dilated lymphatics (asterisks) within the thyroid parenchyma show endothelial cells stained with D2-40; the follicular cells are negative. (b) Follicular epithelial cells and colloid material are positive for CK-7 (arrows); lymphatic channels (asterisks) are nonreactive ((a) D2-40, (b) CK-7. Scale bar = 50 *μ*m).

As expected, D2-40 monoclonal antibody stained ELCs in the thyroid lymphangioma, but not the follicular epithelial cells which, in turn, showed a positive reaction for CK-7 monoclonal antibody (Figure 1). In the PL, MCs were positive for the both markers. The histopathological and immunohistochemical findings on sections from PL of each group are then presented.

#### 3.2.1. Control Group

**Figure 2:**
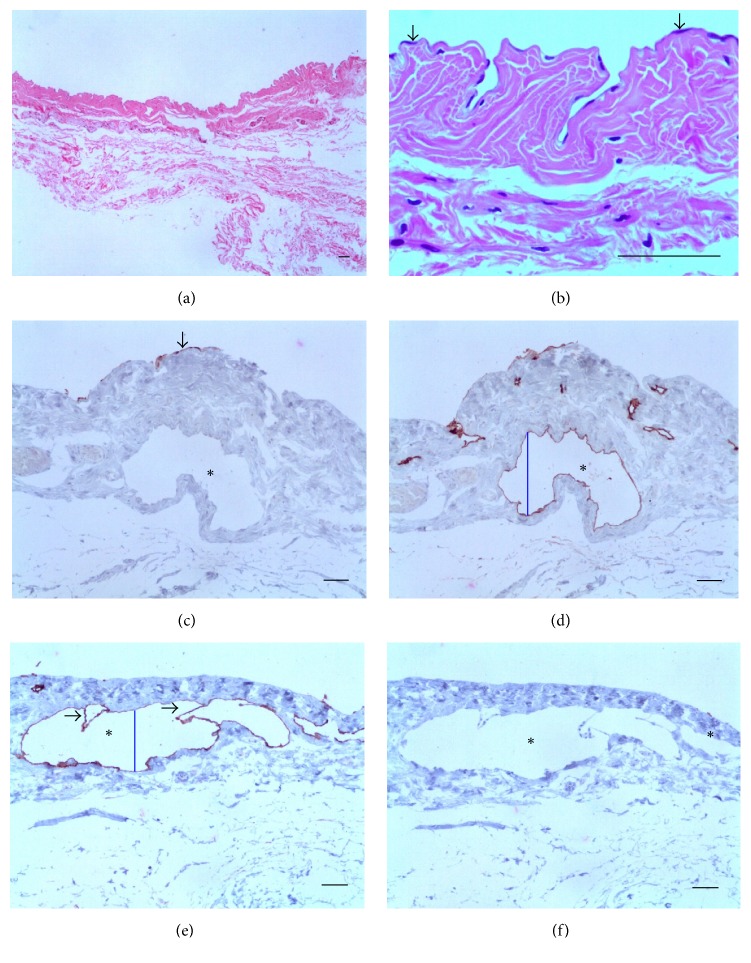
Control group. (a) The PL is thin, with a wavy luminal surface. (b) Monolayer of flat MCs (arrows) without any inflammation below. (c) MCs positive for CK-7. (d) MCs positive for D2-40. (e) Two dilated lymphatics (asterisks) in the submesothelial layer; the valves are clearly seen in the greatest of them (arrows). The transverse diameter (blue line) of the lymphatic is 136.5 *μ*m, and (f) LVs (asterisks) negative for CK-7; no MCs are detected on the free surface ((a), (b) HE; (c), (e) D2-40; and (d), (f) CK-7. Scale bar = 50 *μ*m).

During surgery, no gross inflammatory appearance was observed and up to 0.5 mL of clear serous fluid in testicular vaginal cavity was found. The PL showed a thin wall, lined by a single layer of flattened MCs (Figure 2(a)). In higher magnification, the luminal surface was wavy (Figure 2(b)). Usually, however, the surface was flat and MCs were absent (Figures 2(e) and 2(f)). No inflammatory cells were detected in the submesothelial. LVs were dilated (Figures 2(e) and 2(f)). Additional data of this case, 17-year-old patient; 0 Mf/11 mL of blood; three female AWs, were obtained from LVs of the spermatic cord. No lymphatic fistula was observed per surgery in spite of diffuse intrascrotal lymphangiectasia.

#### 3.2.2. Group with Dead AWs

**Figure 3:**
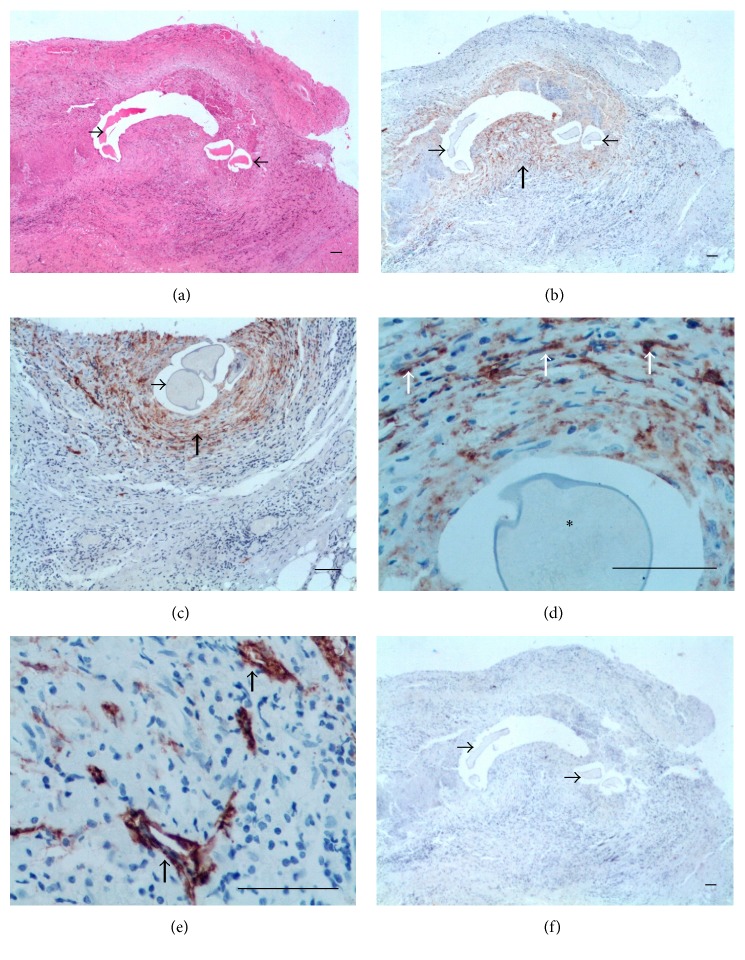
Group with dead AWs in the PL. (a) Dilated LVs containing granulomas surrounding remains of AWs (arrows). ((b) and (c)) Rim of spindle cells positive for D2-40 compounding the granulomas (thick arrows) around remains of degenerated adult worm (thin arrows). (d) Higher magnification of the center of a filarial granuloma exhibiting D2-40 positive cells (arrows) surrounding remnant of degenerated adult worm (asterisk). (e) Small LVs at the periphery of the granulomas (arrows). (f) No cells positive for CK-7 either in surface or forming the granuloma; remnants of the worm are pointed out (arrows) ((a) HE; (b), (c), (d), (e) D2-40; and (f) CK-7. Scale bar = 50 *μ*m).

The PL was thicker when compared to that of living AWs carriers and exhibited a diffuse, chronic inflammatory infiltrate. In addition, lymphatics were occupied by granulomas around remains of AWs in different stages of disintegration (Figure 3(a)). Numerous elongated cells, positively stained for D2-40, were identified in the granulomas, arranged in concentric layers around parasite remains (Figures 3(b), 3(c), and 3(d)). At the periphery of granulomas, apparently newly formed microvessels with diameters varying from 3.0 *μ*m to 4.2 *μ*m (mean, 3.6 *μ*m) stained positively for D2-40 (Figure 3(e)). No staining for CK-7 was observed and MCs were not identified either in the granulomas or on the free surface of the parietal layer (Figure 3(f)). Additional data of this case are as follows: a 20-year-old patient who denied antifilarial treatment before the first consultation; the total fluid volume was 1.0 mL; 175 Mf/mL; positive ipsilateral AWs nests in the spermatic cord were seen. The physical examination before DEC treatment showed a small, thick paratesticular area that the histological examination proved to be a PL nondrug related nodule containing remains of degenerated AWs. After DEC therapy, the patient developed two nodules in the spermatic cord. No lymphatic fistula was observed during surgery in spite of diffuse intrascrotal lymphangiectasia.

#### 3.2.3. Acute Filarial Hydrocele Group

**Figure 4:**
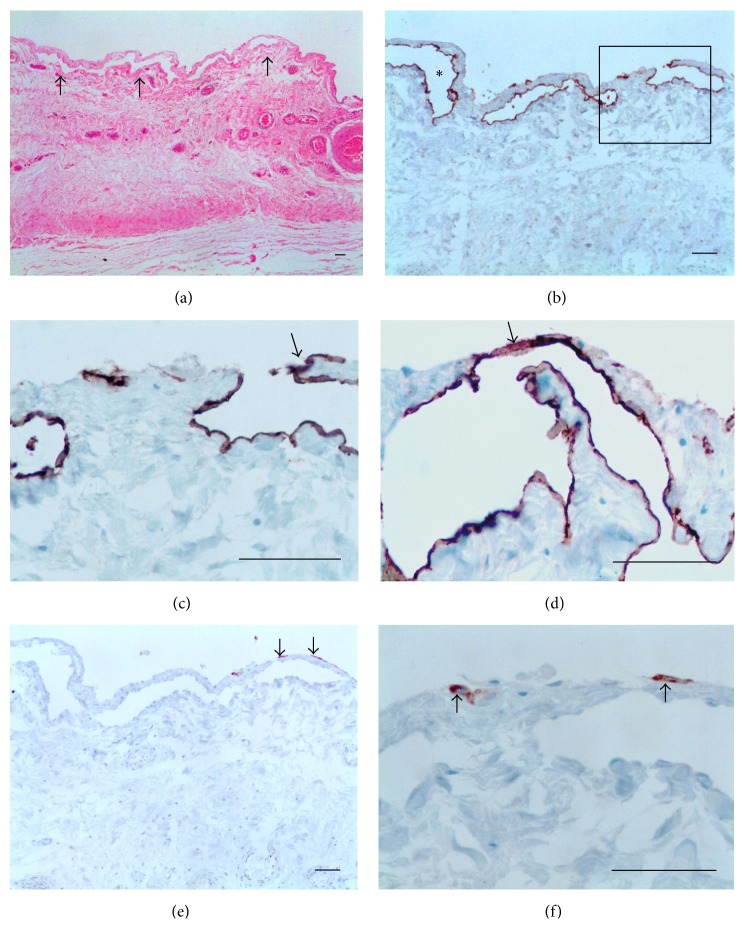
Acute hydrocele. (a) The wall is edematous and contains submesothelial lacunae (arrows). Inflammatory cells are absent. (b) D2-40 stain demonstrates that the lacunae (asterisk) are lined by ELCs. The box displays a stoma communicating a lymphatic lacuna to the mesothelial surface. (c) Higher magnification of area shown in the box: ELCs form a continuum with the mesothelial layer (arrow). (d) ELCs too close to the surface (arrow). (e, f) CK-7 positive only for superficial mesothelial cells (arrows) ((a) HE; (b), (c), (d) D2-40; and (e), (f) CK-7. Scale bar = 50 *μ*m).

The cases included one nondrug related and two after DEC intake after consultation. The PL showed edema, but no inflammatory cells. True lymphatic lacunae with diameter ranging from 28.9 *μ*m to 89.3 *μ*m (mean, 54.4 ± 22.7 *μ*m) were found in parallel to the mesothelial layer (Figures 4(a), 4(b), and 4(c)) and, in some areas, the LECs were located in close proximity to the mesothelial layer with little intervening stroma (Figure 4(d)). In one case, a well-identified small opening with 23 *μ*m in diameter which linked the lacunae to the luminal surface (Figures 4(b) and 4(c)) was seen. The diameter of deeper lymphatics (not shown) varied from 53 *μ*m to 226 *μ*m (mean, 120 ± 66 *μ*m). Positive immunohistochemistry for CK-7 was only observed in superficial MCs (Figures 4(e) and 4(f)). Additional data of the case in Figure 4 are as follows: 16-year-old patient; the total fluid volume was 9.0 mL; 5 Mf/11 mL. The nodule, previously discovered 11 days at the supratesticular area, was nondrug related and was excised 20 days after the beginning of patient discomfort. A mild diffuse lymphangiectasia was observed during surgery.

#### 3.2.4. Filaricele Group

This group was composed of patients without an earlier history of acute filarial hydrocele, but with lymphatic fistulae detected during surgery. In this group, besides lymphangiectasia, another important finding was reactive mesothelial hyperplasia classified from grade 0 to grade 3. In general, the higher was the degree of the reactive process, the more complex were the structural arrangements of the MCs, and the more severe were the fibrosis, edema, and the inflammatory process.

**Figure 5:**
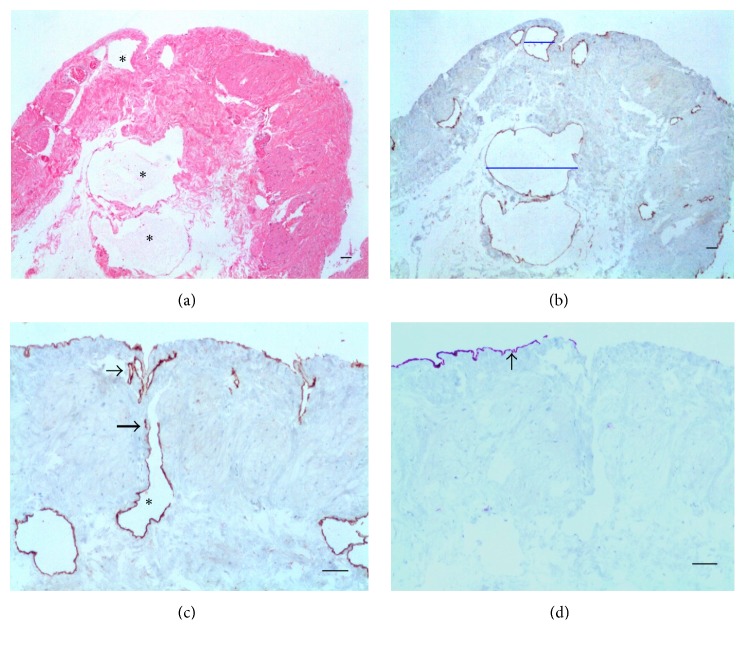
Filaricele grade 0. (a) Dilated LVs (asterisks) pat different levels of the wall. No inflammatory activity is observed. (b) Transverse diameters (blue lines) of the LVs are, respectively, 136.2 *μ*m and 438.2 *μ*m. (c) Lymphatic (asterisk) receiving a branch (thick arrow) from smaller and more superficial vessels (thin arrow). (d) MCs are seen only on the luminal surface (arrows) ((a) HE; (b), (c) D2-40; and (d) CK-7. Scale bar = 50 *μ*m).

*(1) Filaricele Grade 0 Subgroup (4 Cases)*. The cases revealed dilated lymphatics at different levels of the PL with the more superficial ones very near to the luminal surface (Figures 5(a) and 5(b)). Positive immunohistochemistry for CK-7 was only observed in superficial MCs (Figures 5(c) and 5(d)). No inflammatory activity was observed. Additional data of the case in Figure 5 are as follows: 26-year-old patient; the total fluid volume was 5.6 mL; 1,371 Mf/mL; AWs nests nonsensitive to DEC were seen by ultrasound located at supratesticular area and were removed during surgery, when diffuse lymphangiectasia was also observed.

**Figure 6:**
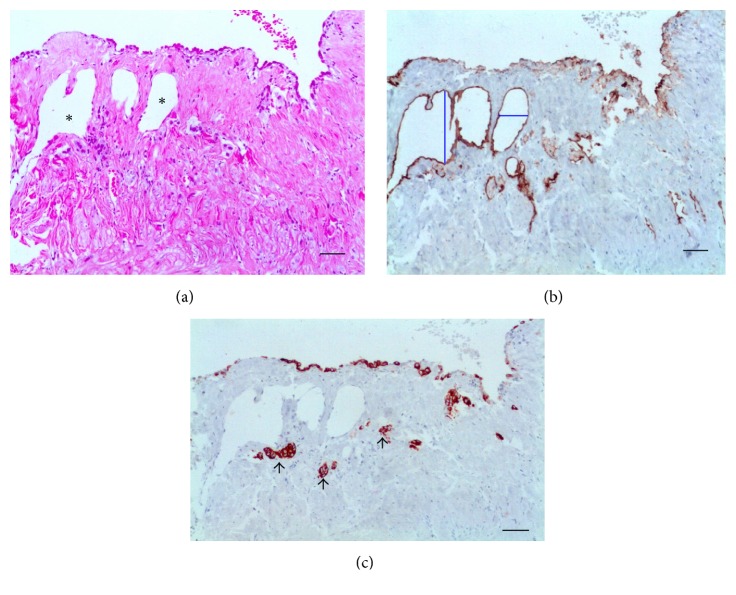
Filaricele grade 1. (a) Mild, focal lymphocyte infiltrate. Dilated submesothelial LVs (asterisks). (b) Transverse diameters (blue lines) of the l LVs are, respectively, 130.8 *μ*m and 56.7 *μ*m. (c) Cuboidal MCs both on the surface and forming small blocks (arrows) ((a) HE, (b) D2-40, and (c) CK-7. Scale bar = 50 *μ*m).

*(2) Filaricele Grade 1 Subgroup (5 Cases)*. The lymphatics were dilated and no inflammatory infiltrates were seen (Figures 6(a) and 6(b)). Cuboidal MCs were found in both the luminal surface and small blocks forming in the most superficial area of the wall (Figure 6(c)). Additional data of the case in Figure 6 are as follows: 37-year-old patient; the total fluid volume was 138 mL; 0 Mf/11 Ml. No AWs nest was identified by ultrasound before surgery. A diffuse lymphangiectasia was observed during surgery. The patient had a previous history of antifilarial treatment at least a decade ago before his first consultation at NEPAF. He did not remember to have had any local adverse reaction after taking the pills.

**Figure 7:**
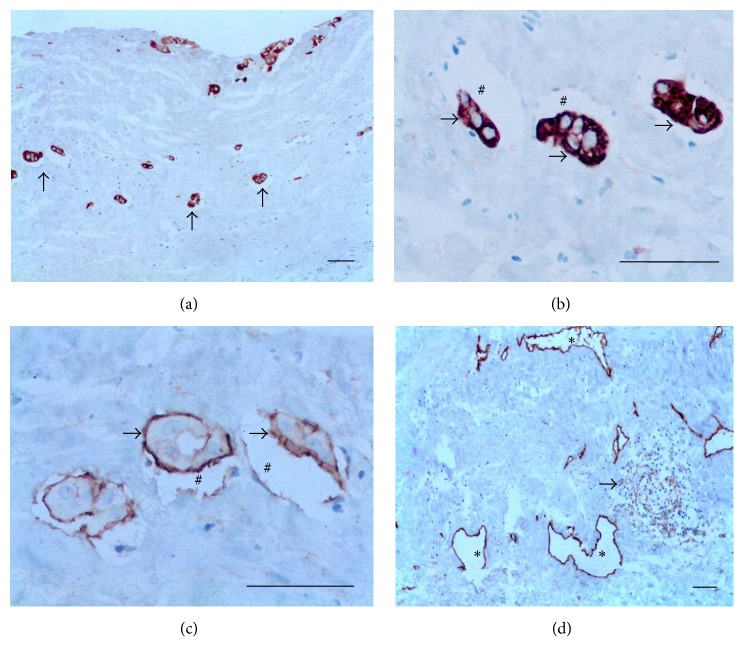
Filaricele grade 2. (a) Blocks of MCs (arrows) reach more than a third of the wall and are arranged in rows parallel to the luminal surface. (b) Cytoplasm of MCs (arrows) positive for CK-7; the spaces (**#**) are retraction artifacts. (c) Membrane pattern of D2-40 staining of MCs (arrows); retraction artifacts (#). (d) Mild, focal infiltrate of lymphocytes (arrow) among deep LVs (*∗*): transverse diameters vary from 21.2 *μ*m to 64.4 *μ*m (mean: 45.6 *μ*m) ((a), (b) CK-7; (c), (d) D2-40. Scale bar = 50 *μ*m).

*(3) Filaricele Grade 2 Subgroup (6 Cases)*. Hyperplasic MCs reach two-thirds of the wall thickness and show a tendency to arrange in rows parallel to the luminal surface (Figure 7(a)). Reactive mesothelial blocks were entrapped within fibrous tissue, with retraction artifact (Figures 7(b) and 7(c)). A mild, focal infiltrate of lymphocytes was observed near lymphatics (Figure 7(a)). Additional data from the case in Figure 7 are as follows: 18-year-old patient; the total fluid volume was 59 mL; 0 Mf/11 mL. No AWs nest was identified by ultrasound before surgery. A diffuse lymphangiectasia was observed during surgery.

**Figure 8:**
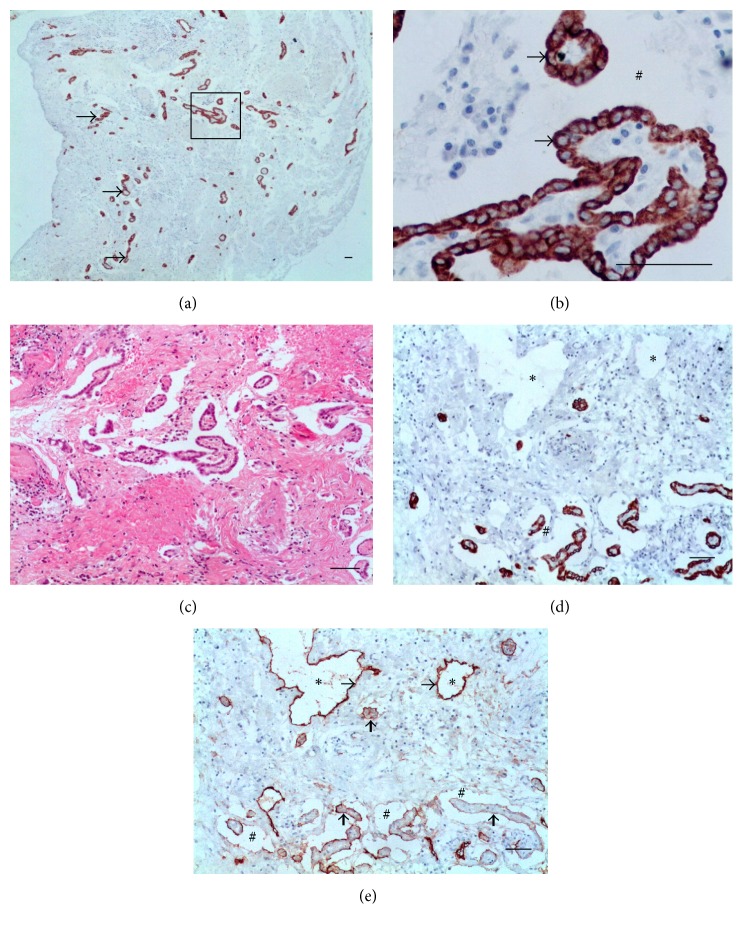
Filaricele grade 3. (a) MCs pervade the whole width of the PL and form complex gland-like or papillary structures (box). MCs arranged in rows parallel to the free surface (arrows). (b) Papillary structure shown in the box. The mesothelial cells have an epithelial appearance and bland nucleus without atypia (arrows). Retraction artifacts (#). (c) Diffuse infiltration of macrophages and lymphocyte. (d) Retraction artifacts (#) should not be mistaken as lymphatics (asterisks), which do not stain for CK-7. (e) Dilated LVs (asterisks) lined by a single layer of ELs (thinner arrows); in this area, transverse diameters of the lymphatics varied from 43.4 *μ*m to 112.9 *μ*m. MCs (thicker arrows) and retraction artifacts (#) ((a), (c), (d) CK-7; (b) HE; and (e) D2-40. Scale bar = 50 *μ*m).

*(4) Filaricele Grade 3 Subgroup (5 Cases)*. The hallmark of this subgroup was a marked reactive hyperplasia of MCs affecting the whole thickness of the PL. The MCs formed rows parallel to the surface (Figure 8(a)). The mesothelial proliferation had an epithelial appearance with complex structural patterns that took the form of gland-like or papillary structures, but without cellular atypia (Figure 8(b)). The matrix was edematous and had a diffuse infiltrate of inflammatory lymphocytes (Figure 8(c)). Retraction artifacts were frequently seen around the blocks of the MCs and should not be interpreted as lymphatic invasion (Figure 8(d)). The lymphatics were tortuous and dilated (Figure 8(e)). Additional data of the case are as follows: 22-year-old patient; the total fluid volume was 57 mL; 0 Mf/11 mL. No AWs nest was identified by ultrasound before the surgery. A diffuse lymphangiectasia was observed during surgery at supra- and infratesticular areas. The patient did not suspect an increased volume of the scrotum and did not have a previous history of antifilarial treatment.

#### 3.2.5. Chylocele Group (4 Cases)

**Figure 9:**
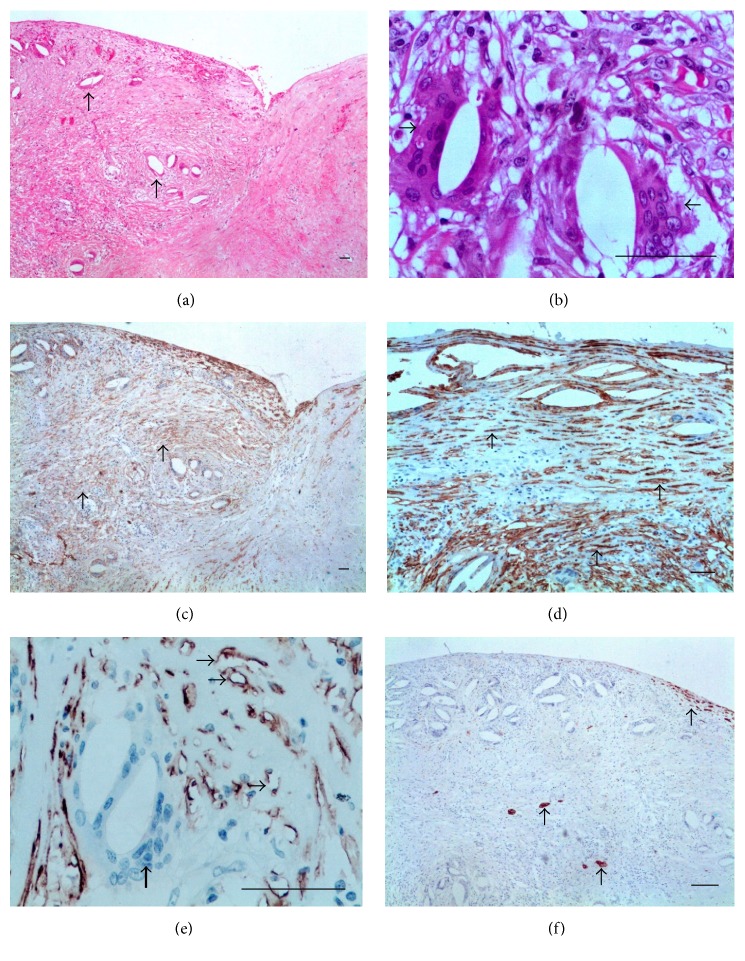
Chylocele. (a) PL thickened with numerous granulomas (arrows) surrounding spindle-shaped clefts of cholesterol crystals. (b) Granulomas with foreign-body giant cells (arrows) encasing cholesterol crystals (the cholesterol disappeared during tissue processing, leaving an empty cleft). (c) Disperse immunoreactivity for D2-40 in areas rich granulomas (arrows). (d) Spindle cells stained by D2-40 (arrows). (e) Microvessels stained by D2-40 (arrows) with diameters varying from 3.6 *μ*m to 5.7 *μ*m (mean, 4.5 *μ*m). The giant cells (thick arrow) are negative for D2-40. (f) MCs (arrows) located at different levels ((a), (b) HE; (c), (d), (e) D2-40; and (f) CK-7. Scale bar = 50 *μ*m).

Patients with lymphatic fistulae generated a milky fluid detected during surgery. The case with more severe lesions showed fibrosis and large numbers of optically empty and spindle-shaped clefts representing cholesterol crystals (the cholesterol dissolves during tissue processing), surrounded by small granulomas with foreign-body giant cells, histiocytes, and lymphocytes (Figures 9(a) and 9(b)). A strong and diffuse immunoreactivity to D2-40 was observed in spindle cells localized all-around the cholesterol granulomas (Figures 9(c) and 9(d)). Microvessels stained with D2-40 were also noticed (Figure 9(e)). CK-7 immunohistochemical stain did not show any overlap with the D2-40 positive spindle cells, being positive only for small cluster of MCs localized more deeply (Figure 9(f)), typifying a grade 3 used filaricele. Additional data of this case are as follows: 58-year-old patient; the total fluid volume was 140 mL; 0 Mf/11 mL. No AWs nest was identified by ultrasound before surgery; the testicle presented important alteration before surgery and an orchiectomy was performed during the chylocele repair. A diffuse and severe supra- and paratesticular lymphangiectasia was observed during surgery.

## 4. **Discussion**

The mesothelium, the basal lamina, and the submesothelial connective tissue stratum are the three basic components of the serous membranes. The submesothelial stratum includes variable amounts of collagen, elastic fibers, fibroblast-like cells, arteries, veins, and lymphatic vessels [21]. The tunica vaginalis testis consists of visceral, extending over the testis and epididymis, and the parietal layer (PL), lining the internal scrotal wall. They are normally separated by a few milliliters of fluid [22]. The lymphatic network of the PL consists of superficial and deep plexuses, both being confined to the connective stratum of the tunica; superficial LVs are intimately related to the mesothelial lining. There are no lymphatic plexuses in the visceral layer [23]. Submesothelial terminal lymphatics form specialized drainage units called lacunae. The single layer of mesothelial cells of the PL is interrupted by stomata which connect directly submesothelial lacunae with the vaginal cavity of the testis [24]. The superficial plexus communicates by narrow channels with the deep plexus which lies within the deeper portion of the basal fibrous layer [23].

This study, based on a group of very well-studied patients, focuses on the LECs and their possible role in the pathogenesis of BF. Lesions of the PL varied greatly in type and severity but, regardless of that, lymphangiectasia is the central alteration, while the presence of alive or dead AWs at that location is rare, as sustained by Dreyer et al. [4]. The lymphangiectasia seems to affect all levels of the intrascrotal lymphatic network, including lymphatics of the PL. Lymphangiectasia was evidenced even before abnormal fluid accumulation in testicular vaginal cavity and in the absence of inflammatory process or fibrous thickening in the PL (Figure 2(b)). So, it is likely that lymphangiectasia is the earliest lesion in the genesis of filaricele.

In our surgical [12] and pathological [25] experience, AWs in the PL lymphatics, whether living or dead, were found only in two cases, one of which is included in the present study. Therefore, the PL lymphatics appear to be the less preferred intrascrotal location of AWs and their absence does not rule out the filarial etiology of fluid accumulation in the tunica vaginal cavity. From this case, however two particularly interesting features can be gleaned: the unprecedented identification of D2-40-labeled cells composing the granuloma cell population (Figures 3(b), 3(c), and 3(d)) and the presence of small, apparently newly formed lymphatics in the periphery of granulomas (Figure 3(e)). As D2-40 is also expressed in other normal and tumor nonendothelial cells, including dendritic cells [26], which are involved in granuloma formation in some conditions as in tuberculosis, the hallmark of granulomatous disease [27], the ratification of the ELCs in this case would require the use of a panel with different markers. In spite of this, it is reasonable to speculate that at least some of D2-40-labeled cells present in the filarial granulomas really originate from the lymphatic endothelium. In fact, this finding would be hardly a surprise for an intravascular parasite. In this regard, parallels can be drawn with some studies in mansoni schistosomiasis. It has been shown, for instance, that blood vessel endothelial cells take part in the development of hepatic periovular granuloma in murine mansoni schistosomiasis [28]. Proliferation of tiny blood capillaries was also evidenced at the early stages of granuloma formation in schistosomiasis, gradually decreasing thereafter, older granulomas becoming almost avascular structures [29]. It is worth mentioning that human ELCs, when exposed to filarial antigens, live parasites* (Brugia malayi)* or infected patient serum, induce both significant proliferation and differentiation into tube-like networks* in vitro *[30]. AWs also release excretory-secretory products that induce human blood cells, specifically monocytes, to produce lymphangiogenic factors such as IL-8 and VEGF-A, which stimulate the formation of lymphatic vessels* in vivo *[31]. Because the endothelium (be it vascular or lymphatic) is closely associated with cells mediating immune responses and inflammation, efforts have been targeted at understanding the interaction between LECs and the filarial parasites [32, 33].

A remarkable finding was the identification of lymphatic stomata in acute filarial hydrocele (Figure 4). Only recently, they were unequivocally identified for the first time on the PL of the tunica vaginalis of humans by Wang et al. [24]. Under physiological conditions, they open and close by turns. The closed lymphatic stomata may represent a reserve capacity of absorption in serous cavities. An excessive accumulation of fluid in the serous cavities could lead to stomata opening, resulting in the absorption of excess fluid. The opening and closing of the lymphatic stomata can be regulated by some cytokines. Fluid accumulation in testicular vaginal cavity may enhance the demand for fluid absorption, which causes the closed lymphatic stomata to open and accelerate the lymphatic drainage of the fluid [24]. The stoma identified was 23 *μ*m in diameter (Figure 4(c)), comparing to a 1-2 *μ*m in normal conditions [24]. It is, therefore, conceivable that the transitory lymphatic obstruction by nodule formation in the spermatic cord resulting in an impairment entrance of serous fluid into the lymphatic network [11] could widen the stomata. It should be emphasized that surgical specimens of the PL from patients with acute hydrocele are very rare. To our knowledge, only three cases, associated with formation of filarial intrascrotal nodules, underwent surgical repair; all of them are included in this study. It is possible that more stomata would be found in this and in the other two cases if a more extensive survey with a greater number of samples had been performed. Besides, the more intense are the inflammation, fibrosis, and mesothelial hyperplasia, the more difficult is the identification of the stomata. This may explain, in principle, why they are not seen in cases of filaricele, which form the majority of cases in the present investigation, and in chylocele, associated or not with haematocele. The role of the stomata in the chronic hydrocele pathogenesis, whether or not filarial, filaricele, and chylocele, remains a challenging task.

Norões and Dreyer [12] consider that filaricele is the prototype of the chronic condition occurring in endemic areas, referred to in the literature as “chronic filarial hydrocele,” “chronic hydrocele,” or simply hydrocele. Among patients in this group, the histological features were more heterogeneous and the submesothelial lymphatic network tended to disorganize with increasing severity of lesions such as edema, inflammatory infiltrate, and mesothelial hyperplasia. This is corroborated when comparing the cases situated on the extremes of the spectrum. In the case with grade 0 filaricele (Figure 5), the PL was very thin, without inflammatory infiltrate or fibrosis, and the MCs were confined to the free surface. Cases of grade 3 filaricele, on the other hand, showed a very thick PL, with an intense mesothelial hyperplasia affecting the entire wall thickness (Figure 8(a)). In cases in which the PL is not so thick, dilation is clearly seen in all levels of the lymphatic network (Figures 5 and 6). Probably the severity of lesions is associated mainly with the fluid composition (including also secretory-excretory antigen from AWs) or time of evolution and not with the volume, as earlier suggested by Norões and Dreyer [12]. Although no lymphatic fistula could be identified histologically, leaking lymph fluid from ruptured lymphatics is an outstanding event observed* in vivo* during surgeries [12] and can be witnessed in video [https://www.youtube.com/watch?v=otJMqYlHkKs].

The severe reactive mesothelial processes may be very histologically worrisome. Gland-like formation with outward branches, as described in Figures 8(a), 8(b), and 8(c), is an additionally cofounding finding, because they can be seen in true epithelioid mesotheliomas or adenocarcinomas. Thus, the threshold for a diagnosis of malignancy must be set higher in hydroceles, filarial or not. A histological diagnostic clue can be helpful: the proliferating MCs in benign reactions often form lines that are parallel to the surface of the PL [34], a pattern observed in filaricele.

MCs are fundamental to the maintenance of serosal integrity and homeostasis and play a critical role in normal serosal repair following injury. MCs secret inflammatory and profibrotic mediators, differentiate, and migrate into the injured tissues where they contribute to fibrogenesis [35]. This is fully in connection with the driving concept of the pathogenesis of filaricele: an accumulation of protein-rich, nonmilky lymph fluid in the testicular vaginal cavity due to ruptured dilated lymphatics [12]. The aggressive capacity of nonmilky lymph on mesothelial seen in filaricele can be enhanced by the presence of chylomicrons, as shown in Figure 9.

A particularly intriguing finding in this case was the presence of numerous D2-40-positive spindle cells arranged in bundles (Figures 9(c) and 9(d)). It has demonstrated that D2-40 labels myoepithelial cells in a variety of breast lesions [36]. Importantly, tissue activated myofibroblasts have been identified as the cells responsible for the establishment and progression of the fibrotic process and can originate from several sources including various cells such as endothelial cells. However, the role of the phenotypic transition of endothelial cells into mesenchymal cells (endothelial to mesenchymal transition or EndoMT) in the pathogenesis of fibrotic disorders has not been fully elucidated [37]. This issue certainly holds great importance not only in hydrocele, but also in the different clinical manifestations of filariasis, in which fibrosis is an important component. The newly formed lymphatic microvessels (Figure 9(e)) raise other questions. According to Semo et al. [3], little is known currently about the identity of the cells contributing to the formation of new LVs. Based on the results obtained from the developmental studies, they speculate that new LECs could arise from preexisting lymphatic vessels through the process of lymphangiogenesis or, alternatively, they could originate from angioblast niches maintained within veins, or throughout the body, via lymph vasculogenesis. On the other hand, cells might transdifferentiate in situ from surrounding mesenchymal cells.

## 5. Conclusions

The present investigation covered very well-characterized clinical presentations of intrascrotal BF; some of them are seldom diagnosed even in a research setting, such as the acute filarial hydrocele. Some standpoints should be underlined: namely, (a) the identification of lymphatic stomata which is reported in filarial acute hydrocele for the first time; (b) the rarity in the AWs in PL, in spite of marked lymphangiectasia and the absence of Mf in the fluid from testicular tunica vaginalis cavity; (c) the apparent involvement of ELCs in filarial granuloma; (d) neoformation of lymphatic capillaries in filarial granuloma and in chylocele; and (e) the possible phenotypic transition of LECs into myofibroblasts in chylocele. Taken together, these data suggest that, far from being bystanders, LECs have shown to play an active role in the interplay between lymphatic vessels and the filarial parasites. The issues still open may inspire further investigations in different endemic areas in order to better understand the role of LECs in the pathogenesis of hydroceles/filariceles and of other clinical presentations of lymphatic filariasis.

## Figures and Tables

**Table 1:** General characteristics of the patients.

Groups	*N* (%)	Mean (range) age (years)	Mean (range) fluid (mL)	Geometric mean (range) Mf density	
				1 mL	11 mL
Control	4 (12.5)	19 (17–21)	0.32 (0.4–0.2)	501.0 (102–2596) (*N* = 3)	Negative^*∗*^ (*N* = 1)
Dead AWs	1 (3.2)	20 (20-20)	1.0 (1.0-1.0)	175 (175-175) (*N* = 1)	NA^*∗∗*^
Acute hydrocele	3 (9.4)	20 (16–23)	10.5 (9.0–12.3)	327 (327-327) (*N* = 1)	3.9 (3–5) (*N* = 2)
Chylocele	4 (12.5)	41.7 (30–58)	134.9 (99.5–157)	79 (79-79) (*N* = 1)	Negative (*N* = 3)
Filaricele	20 (62.5)	26.7 (18–37)	85.7 (5.6–191.0)	33.0 (1–1371) (*N* = 14)	1 (1-1) (*N* = 1); Negative (*N* = 5)
Total	32 (100.0)	26.8 (16–58)	71.5 (0.2–191)	63.2 (1–2596) (*N* = 20)	2.5 (1–5) (*N* = 3); Negative (*N* = 9)
Filaricele, grades^*∗∗∗*^					
0	4 (20.0)	22 (18–26)	8.0 (5.6–11.1)	304.5 (25–1371) (*N* = 4)	NA^*∗∗*^
1	5 (25.0)	30 (25–37)	93.0 (53.3–138)	24.6 (2–425) (*N* = 4)	Negative (*N* = 1)
2	6 (30.0)	26.8 (18–33)	116.4 (58–191)	14.6 (1–196) (*N* = 4)	Negative (*N* = 2)
3	5 (25.0)	27 (22–34)	103.8 (57–148)	25.2 (5–127) (*N* = 2)	(1-1) (*N* = 1); Negative (*N* = 2)

^*∗*^Negative: count = 0; ^*∗∗*^NA = not applicable. ^*∗∗∗*^Grades' percentage base = 20.
